# Sex Trafficking Related Knowledge, Awareness, and Attitudes among Adolescent Female Students in Nepal: A Cross-Sectional Study

**DOI:** 10.1371/journal.pone.0133508

**Published:** 2015-07-15

**Authors:** Roman Shrestha, Pramila Karki, Asha Suwal, Michael Copenhaver

**Affiliations:** 1 Department of Community Medicine & Health Care, University of Connecticut Health Center, Farmington, Connecticut, United States of America; 2 Center for Health, Intervention, and Prevention, University of Connecticut, Storrs, Connecticut, United States of America; 3 Department of Nursing, Nobel College, Sinamangal, Kathmandu, Nepal; 4 Department of Allied Health Sciences, University of Connecticut, Storrs, Connecticut, United States of America; Leibniz Institute for Prevention Research and Epidemiology (BIPS), GERMANY

## Abstract

**Background:**

Sex trafficking has been a long-standing concern in Nepal. Very little has been achieved, however, in terms of actual reduction in the number of victims despite numerous anti-sex trafficking programs. This situation may be attributable to a lack of empirical evidence upon which to formulate anti-sexual trafficking interventions. This study aimed to assess sex trafficking-related knowledge, awareness and attitudes, and factors associated with sex trafficking awareness and attitudes towards the victims of sex trafficking and/or anti-sex trafficking campaigns among adolescent female students in Nepal.

**Methods:**

A cross-sectional study was conducted between August–September 2013 among 292 adolescent female students (>10 years old) using systematic random sampling from three high schools in Sindhupalchowk district, Nepal. As an initial step, descriptive analyses were employed to characterize the data and multivariate logistic regression analyses were used to explore factors associated with sex trafficking awareness and related attitudes.

**Results:**

Seventy-six percent of sampled students reported that they were aware of sex trafficking and 94.6% indicated media (i.e., radio or television) as the primary sources of their knowledge. Fifty-one percent mentioned relatives/friends as mediators of sex trafficking, 60.4% reported promise for better jobs as the primary attraction behind sex trafficking, and 48.6% mentioned adolescent females as the most vulnerable group for sex trafficking. Over half (56.8%) of the respondents had positive attitudes towards the victims of sex trafficking and/or anti-sex trafficking campaigns. Age (OR = 3.38, 95% CI:2.51–4.55), parents’ occupation (OR = 3.89, 95% CI:1.58–9.58), and having a radio/TV at home (OR = 6.67, 95% CI:3.99–9.54) were significantly associated with awareness, whereas being younger (OR = 0.67, 95% CI:0.55–0.79) and having joint-family (OR = 2.67, 95% CI:1.49–4.80) were significantly associated with having a positive attitudes towards the victims of sex trafficking and/or anti-sex trafficking campaigns.

**Conclusion:**

Findings presented have important implications for anti-trafficking programs, in particular those designed to educate the adolescent females who are at most-risk of sex trafficking. Educational programs need to include specific interventions to improve knowledge and attitudes towards sex trafficking among adolescent females in Nepal.

## Introduction

Trafficking in persons, especially children and young women, is a reflection of many of the complex social issues existing in the global society. In recent decades, however, a rising concern about violence against women (VAW) worldwide has put human trafficking on the international agenda. In view of the growing magnitude of the problem and its link with the commercial sex industry, coercive labor, HIV/AIDS pandemic and other forms of human rights violations has added urgency to global anti-trafficking movements [1].

Human trafficking is a gross violation of human rights and a serious crime that inhumanly abuses women, children, and men for numerous purposes, most commonly for sexual exploitation and forced labor [2]. The United Nations defines human trafficking as:

“…the recruitment, transportation, transfer, harbouring or receipt of persons, by means of threat or use of force or other forms of coercion, of abduction, of fraud, of deception, of the abuse of power or of a position of vulnerability or of giving or receiving of payments or benefits to achieve the consent of a person having control over another person for the purposes of exploitation [3].”

The United Nation Office on Drugs and Crime (UNODC) reports that the official estimates of the number of people who are trafficked each year vary significantly from tens of thousands to millions [2]. Such huge variations in estimates are due to the covert nature of the crime and the several procedural difficulties in assembling information on these issues. Regardless, the UNODC report estimated that women accounted for over half of all trafficking victims (i.e., 55–60%) detected worldwide. Possibly most alarming of all is a rise in the number of child victims in the recent years. The same report revealed that the number of identified child victims of human trafficking globally has gone up by 7% in a three year period: 27% between 2007–2010 as compared to 20% between 2003–2006 [2].

Trafficking in women and girls, primarily subjected to sexual exploitation and forced labor, has been a long-standing concern in Nepal. Within Nepal, the victims are often trafficked from rural areas to the urban centers for sexual exploitation in places such as dance restaurants, massage parlors, carpet and garment factories, brick-kilns and others [4, 5]. The Trafficking in Persons Report compiled by the US State Department viewed Nepal as one of the “source” countries in the Asian network of trafficking. Typical transit for Nepali victims of trafficking are India, Pakistan, the Middle East, and other countries such as Malaysia [4]. Although the actual magnitude of trafficking in persons from Nepal is unknown, some published figures suggest that between 5,000 to 7,000 Nepali women and girls are trafficked to India alone each year [6]. Furthermore, the UNODC Global Report in Trafficking 2012 estimated that child victims, below age of 16 years, accounted for 36% of all trafficking victims in Nepal [2].

Trafficking in persons in Nepal is a very complex and multi-causal phenomenon. Specifically, the interaction of poverty, gender, education, age, and relevant policy deeply affect the vulnerability of women and children to trafficking. Perry and McEwing reported social determinants (i.e. familial dispute, violence, innocence and ignorance, illiteracy, and various forms of discrimination) to be the major causes behind trafficking. As one of the poorest and least developed countries in the world, Nepal currently ranks 157^th^ out of 187 countries on the Human Development Index (HDI) [7]. Similarly, the literacy rate is 65.9%, with a large proportion of the population being unemployed (42%) and living below poverty line (38%) [8]. Such high rates of unemployment and economic insecurity, poverty, coupled with low educational background and gender inequality are the biggest contributing factors to women and girls being the victims of trafficking in the region [9–11].

Mounting concern over the trafficking of girls and women in Nepal has spurred the government, as well as various non-governmental organizations (NGOs) and international non-governmental organizations (INGOs), to develop and implement several social, cultural, and economic interventions to address trafficking. For example, several NGOs and INGOs in Nepal, such as Maiti Nepal, ABC/Nepal, Family Planning Association of Nepal (FPAN), Child Workers in Nepal (CWIN), and Women’s Rehabilitation Centre (WOREC) have focused on programs that aim to prevent the trafficking of girls and women as well as to rehabilitate survivors and reintegrate them into their communities. Along the same path, the Government of Nepal (GoN) formed the Ministry of Women, Children and Social Welfare (MoWCSW) in 1998, which worked in conjunction with the International Programme on the Elimination of Child Labour (IPEC) and the International Labour Organisation (ILO) to develop a comprehensive thirteen-point strategy for the prevention of Trafficking [10, 12]. In 2007, the GoN took a key step toward addressing this crime by passing the Human Trafficking and Transportation Act (HTTCA), which approves an inclusive legal framework to combat trafficking [12]. Furthermore, the government implemented various national-level policy reforms through the endorsement of the Foreign Employment Policy 2012 and also implemented various interventions under the larger purview of the anti-trafficking framework [12].

Despite the efforts made by the government, NGOs, and INGOs, however, very little has been achieved in terms of actual reduction in the number of trafficking victims. As Kaufman and Crawford pointed out, anti-trafficking programs in Nepal are often designed on the basis of personal judgments by those running the NGOs and little research goes into planning and implementation of prevention efforts. Furthermore, there is also a lack of assessments of knowledge and/or attitudes when designing and implementing programs designed for specific communities or populations [13]. Much of the existing evidence is assembled in NGO publications, which present anecdotes, reports, observation from agencies working to prevent sex trafficking, and policy analysis, rather than the results of empirical research [4, 10, 11, 14–16]. Hence, a clear understanding of individuals who are at most-risk (i.e., girls and young women) about their awareness and attitude towards trafficking is very important to support the development of feasible and effective anti-sex trafficking measures within Nepal and similar areas.

Hence, considering the dearth of information on this important topic, the present study was designed as an initial assessment of the knowledge, awareness, and attitudes of adolescent girls about trafficking. Additionally, this study explored the potential association of socio-demographic variables of adolescent females with their awareness and attitudes related to sex trafficking. We believe that the information obtained from the study will serve as important baseline data in designing relevant and innovative programs for addressing sex trafficking in Nepal, the region with one of the highest rate of trafficking in South-Asia.

## Methods

### Study setting

This cross-sectional study was conducted among high school (i.e., grade 8–12) female students from three high schools in Sindhupalchowk district, Nepal between August and September 2013. Selected district was chosen purposively with the highest rate of trafficking. Sindhupalchowk district, although close to Nepal's capital city Kathmandu, is one of the least developed districts in the country. This region lies within the hilly rugged topography and is relatively poor. According to the Central Bureau of Statistics (CBS) report of 2011 population census, the total population of the district was estimated to be 287,798 and about 45% of them were living below the poverty line. Rural poverty, along with the presence of low literacy rate, has thus increased the migration process in the region, leading to the movement of individuals to seek viable living elsewhere, often in major cities within Nepal (i.e., Kathmandu, Pokhara) or outside Nepal (i.e., India, China, the Middle East) (Central Bureau of Statistics, 2012. Although migration is distinct from trafficking, however, the growing wave of migration has significantly increased in terms of young girls and women being trafficked in the region.

### Sampling method

A list of all female students was obtained from three high schools from the district, which were chosen purposively. All female students who were enrolled in grade 8 through grade 10 were eligible to participate in the study. Based on the observation and history from the school records, students who were less than 10 years old were excluded. Sample size was calculated using the formula

$$n=\frac{Z^{2}.p.\left(1-p\right)}{m^{2}}$$

where,

n = sample size

Z = confidence interval at 95% (standard value of 1.96)

p = estimated prevalence of awareness about sex trafficking

m = margin of error at 6% (standard value of 0.06)

As there were no existing data on awareness and attitudes related to sex trafficking among school students in Nepal, we estimated that 50% of students may have some form of awareness about trafficking. Assuming a non-response rate of 10%, the sample size was estimated to be 294. To obtain our desired target of participants, we decided to recruit a set of number of female students from each school through systematic random sampling.

### Measures and procedure

A structured questionnaire was used to elicit respondents’ awareness and attitudes about sex trafficking. The questionnaire had three sections: 1) socio-demographic characteristics, 2) awareness about sex trafficking, and 3) attitudes towards sex trafficking. Basic socio-demographic variables of the respondents including age, grade (education), marital status, religion, ethnic group, family type (nuclear/joint), family’s primary occupation, family income, educational status of parents, and presence of radio/TV at home were assessed via single self-reported items on the survey.

The dichotomous variable of interest, respondents’ awareness about sex trafficking, was constructed based on responses to the questions, “*Have you ever heard or read about sex trafficking*?*”* Those who answered *“yes”* were classified as being aware about sex trafficking and those who answered *“no”* were classified as being not aware about sex trafficking. Additional questions were asked to assess their knowledge regarding sex trafficking. To evaluate attitude of respondents, we asked them to answer “yes” or “no” to each attitude-related statements. Response of *“no”* to statements, *“Girls and young women who are sex trafficked choose to enter prostitution”*, *“Enough services are available to girls and young women who have been sex trafficked”*, *“Sex traffickers need to sex traffic girls and young women to make a living”*, *“Sex trafficking of girls and young women is not a problem in Nepal”*, *“Government is doing enough to ensure safety of girls and young women against being trafficked”*, *and “Sex trafficked girls and young women who decided to live a normal life are to be looked down upon”*, and response of “yes” to statement *“Girls and young women who have been sex trafficked deserve to be treated with respect”* was considered positive response for each specific statement and vice-versa. We assigned “1” for every positive response in the attitude section and “0” for negative responses, which was based on attitude scale mentioned above created for the purpose of this study. We then summed up the scores to generate an overall score for each respondent. The scores of attitudes was then divided into two categories based on their mean score: those scoring less than mean scores for attitude were classified as “negative” and those scoring equal and more than mean scores were classified as “positive” attitudes.

Before data collection began, the questionnaire was piloted with 5 students, testing for clarity, feasibility and appropriateness for the students. The questionnaire was developed in English, then translated into Nepali language before data collection and finally translated back into English. The researcher briefed about all the jargon used in the questionnaire and provided guidance on how to complete the questionnaire. After briefing, the researcher distributed the self-administered survey questionnaires on paper to eligible students. The students used either pencil or pen to mark their answers on the paper.

The total of 301 female students agreed to participate in the study, however 9 students refused to complete the questionnaire. Therefore, the number of young female adults finally included in the study was 292, giving a response rate of 86.4%.

### Data analysis

Data were entered, cleaned, and analyzed with SPSS software, version 20.0 (IBM Corp., 2011). Descriptive analyses were carried out to characterize the demographics and awareness and attitudes related to sex trafficking, which included numbers and percentages for categorical data and mean (±standard deviation, SD) and range for continuous data. A multivariable logistic regression was conducted to estimate the odds ratio (OR) and 95% confidence interval (CI) for the association of socio-demographic characteristics with awareness and attitudes related sex trafficking. For the purpose of this study, we treated socio-demographic variables as predictor variables and sex trafficking knowledge and attitudes as the outcome variables. All tests were two-tailed, and *p*<0.05 was considered significant.

### Ethical considerations

This study protocol, including the consent procedure, was approved by the Human Research Ethics Committee, Nobel College, Kathmandu, Nepal. Furthermore, the letters of collaboration to conduct this study were obtained from each school and respective Village Development Committees (VDC) in Sindhupalchowk district, Nepal. The students were informed about the purpose of the study and were assured that their responses would be treated confidentially and anonymously. Respondents were also informed that their participation was entirely voluntary and that they were free to decline to answer any question that made them feel uncomfortable. Finally, written informed consent was secured from each study participants and their confidentiality, privacy and anonymity was maintained. For respondents whose age was less than 18 years old, their assent and written informed consent from their parents/guardians were obtained. The questionnaire developed for this study was completely anonymized and did not include any requests for identifying information.

## Results

### Socio-demographic characteristics of the respondents

The socio-demographic characteristics of the participants are shown in Table 1. The mean (SD) age of the respondents was 15.8 (±1.9) years. The number of respondents were evenly distributed between grades. As shown in Table 1, 84.9% of the respondents were unmarried, while 15.1% were married. The large majority of the respondents were Hindu (86.3%), followed by Buddhist (11.6%), and Christian (2.1%). Women from Chhetri and Brahmin ethnic groups accounted for almost two-thirds of the sample (63.1%).

**Table 1:** Sociodemographic characteristics of the respondents (*n = 292*).

Variables	*n*	%
Age (years)		
Mean±SD	15.8±1.9	
Range	12–19	
Grade		
VII	65	22.6
IX	70	24.0
X	68	23.3
XI	48	16.4
XII	40	13.7
Marital Status		
Unmarried	248	84.9
Married	44	15.1
Religion		
Hindu	252	86.3
Buddhist	34	11.6
Christian	6	2.1
Ethnic Group		
Brahmin	72	24.7
Chhetri	112	38.4
Newar	40	13.7
Dalit	18	6.2
Others^a^	50	17.1
Family Type		
Nuclear	136	46.6
Joint^b^	156	53.4
Family’s Primary Occupation		
Agriculture	176	60.3
Business	72	24.7
Daily wages	14	4.8
Others^c^	30	10.3
Family Income (NRS)		
Less than 5,000	66	22.6
5,000–6,999	78	26.7
7,000–9,999	100	34.2
10,000 or above	48	16.4
Parents Educated		
Illiterate	68	23.3
Father only	120	41.4
Mother only	6	2.1
Both	98	33.6
Presence of Radio/TV at Home		
Yes	270	92.5
No	22	7.5

^a^ Sherpa, Tamang, Magar, Rai

^b^ Extended family arrangement

^c^ Transportation, manufacturing, construction, services

Over half of the respondents (53.4%) indicated that they lived in a joint family, while 46.6% lived in a nuclear family. When asked about their family’s primary occupation, the results in Table 1 indicate that 60.3% reported agriculture, 24.7% reported business, 4.8% reported daily wages and 10.3% reported others. Only 16.3% of the respondents reported that their family earned NRS 10,000 (1 USD = NRS 99) or above, whereas almost half of the respondents (49.3%) reported earning less than NRS 7,000. The results show that 41.4% of the fathers of the respondents had formal education compared to 2.1% of their mothers who had no formal education. The majority of the respondents reported to have either a radio or television in their house.

### Sex trafficking knowledge and awareness among the respondents

Of all the respondents, 76% reported that they had heard or read about sex trafficking. The majority of the respondents mentioned media (i.e., radio or television) as their primary source of information about sex trafficking, whereas 49.5%, 36%, 24.3%, and 15.3, respectively, mentioned friends, family/relatives, schools and NGOs/health professionals as sources of information. Relatives and friends were mentioned as mediators for sex trafficking by 51.4% and 34.2% of the respondents respectively. Promise for employment by agent (60.4%) and fraudulent marriage (54.1%) were the primary modes of sex trafficking, followed by fake visits (31.5%) and others modes (10.8%). About 62.2%, 58.6%, 56.8%, and 25.2% of the respondents mentioned poverty, lack of awareness, interest on big money, and illiteracy respectively as reasons for being trafficked (Table 2).

**Table 2:** Sex trafficking knowledge and awareness among the respondents.

Variables	*n*	%
Heard or read about sex trafficking (n = 292)		
Yes	222	76.0
No	70	24.0
Source of information about sex trafficking (n = 222)^a^		
Media	210	94.6
Friends	110	49.5
Family/relatives	80	36.0
School	54	24.3
NGOs/health professionals	34	15.3
Mediators of sex trafficking (n = 222)^a^		
Husband	16	7.2
Relatives	114	51.4
Friend	76	34.2
Agent *(Dalal)*	22	9.9
Modes of sex trafficking (n = 222)^a^		
Employment *(Dalal)*	134	60.4
Fraudulent marriage	120	54.1
Fake visits	70	31.5
Others	24	10.8
Causes of sex trafficking (n = 222)^a^		
Poverty	138	62.2
Lack of awareness	126	56.8
Interest on big money	130	58.6
Illiteracy	56	25.2
Others	22	9.9
Purpose of sex trafficking (n = 222)^a^		
Prostitution	142	64.0
Circus work^b^	44	19.8
Domestic work	74	33.3
Others^c^	6	2.7
Effects of sex trafficking (n = 222)^a^		
Social discrimination	68	30.6
Sexual abuse	26	11.7
Physical abuse	90	40.5
Mental abuse	8	3.6
Age group most vulnerable (n = 222)		
Less than 15 years	20	9.0
15–19 years	88	39.6
20–24 years	76	34.2
25 years or above	38	17.1
Know someone being sex trafficked (n = 222)		
Yes	108	37.0
No	184	63.0

^a^ Multiple response statement

^b^ Performer in circus, such as acrobatics, juggling, dance, rope climbing, etc.

^c^ Massage parlors, bars/strip clubs, escort services, modeling studios, etc.

Almost two-thirds of the respondents (64%) reported the purpose of sex trafficking to be prostitution, whereas 33.3% and 19.8% reported domestic work and circus work respectively (Table 2). Forty-percent of the respondents identified physical abuse as an effect of sex trafficking, whereas 30.6%, 11.7% and 3.6% identified social discrimination, sexual abuse and mental abuses as the effects of sex trafficking. Almost half of the respondents (48.6%) mentioned that adolescents females between the ages of 10 and 19 years are the most vulnerable group for sex trafficking, whereas 51.3% mentioned youth females ages over 20 years to be the most vulnerable group. About 37% of the respondents reported that they know someone who have been sex trafficked.

### Respondents’ attitude towards sexual trafficking

Overall attitudes of the high school female students regarding sex trafficking, and victims of sex trafficking are illustrated in Table 3. More than half of the respondents (56.8%) believed that trafficking of girls and young women for sex is a problem in Nepal. The majority of the respondents exhibited positive attitudes towards girls and women who are sex trafficked, such as they deserve to be treated with respect, they are not to be looked down upon, etc. Less than half of the respondents (44.1%) believed that there are enough services available to girls and young women who have been trafficked. Forty-five percent of the respondents thought government was doing enough to ensure safety of girls and young women against being trafficked and 33.3% reported sex trafficked girls and young women who decided to live a normal life should be looked down upon (Table 3).

**Table 3:** Respondents’ attitude about sexual trafficking (n = 222).

Variables	*N*	%
Girls and young women who are sex trafficked choose to enter prostitution (Yes)	78	35.1
Girls and young women who have been sex trafficked deserve to be treated with respect (Yes)	158	71.2
Enough services are available to girls and young women who have been sex trafficked (Yes)	98	44.1
Sex traffickers need to sex traffic girls and young women to make a living (Yes)	76	34.2
Sex trafficking of girls and young women is not a problem in Nepal (Yes)	96	43.2
Government is doing enough to ensure safety of girls and young women against being trafficked (Yes)	100	45.0
Sex trafficked girls and young women who decided to live a normal life are to be looked down upon (Yes)	74	33.3

Of the 7 questions that addressed attitudes towards the victims of sex trafficking and anti-sex trafficking campaigns, the scores ranged from 1 to 7 (mean score = 4.36, SD±2.28). Accordingly, 56.8% of the respondents scored at or above the mean and were therefore classified as having a positive attitude towards the victims of sex trafficking and/or anti-sex trafficking campaigns (Table 3).

### Factors associated with awareness and attitudes towards sex trafficking

Multivariable logistic regression analysis reports that age, family’s primary occupation, and presence of radio/TV at home were significantly associated with awareness of sex trafficking. Respondents were 3.38 times (2.51–4.55) more likely to be aware about sex trafficking with each additional year of age. Those whose family’s primary occupation was business were 3.89 times (1.58–9.58) more likely to be aware about sex trafficking than those whose family’s primary occupation was agriculture. Similarly, respondents who had radio or television at home were about 6.67 times (3.99–9.54) more likely to be aware about sex trafficking compared to those who did not (Table 4).

**Table 4:** Association of socio-demographic characteristics with awareness and attitude related to sex trafficking.

Variables	Awareness		Attitude^a^	
	OR^b^	95% CI^c^	OR^b^	95% CI^c^
Age^d^	3.38	2.51–4.55	0.67	0.55–0.79
Marital status				
Unmarried	-	-	-	-
Married	1.96	1.12–3.44	0.64	0.29–1.42
Religion				
Hindu	-	-	-	-
Buddhist	1.59	0.63–4.01	1.54	0.58–4.04
Christian	2.94	0.78–8.88	1.12	0.17–7.37
Family type				
Nuclear	-	-	-	-
Joint	1.50	0.88–2.58	2.67	1.49–4.80
Family’s primary occupation				
Agriculture	-	-	-	-
Business	3.89	1.58–9.58	0.75	0.35–1.62
Daily wages	0.47	0.16–1.43	1.37	0.23–8.34
Others	0.53	0.24–1.19	0.29	0.11–0.83
Parents educated				
Illiterate	-	-	-	-
Father only	1.36	0.59–2.48	0.27	0.08–0.86
Mother only	0.93	0.44–1.82	0.39	0.05–3.51
Both	1.78	0.85–3.39	1.12	0.89–1.56
Presence of radio/TV at home	6.67	3.99–9.54	1.03	0.15–6.89

^a^ Level of attitude was defined as “positive” for those scoring ≥ mean, and “negative” for those scoring < mean

^b^ OR = Odds ratio

^c^ CI = Confidence interval

^d^ increase per additional year of age

As shown in Table 4, age and family type were significantly associated with having positive attitude towards victims of sex trafficking and/or anti-sex trafficking campaigns. Respondents were 1.49 times (1.26–1.81) less likely to have positive attitude towards the victims of sex trafficking and anti-sex trafficking campaigns for each additional year of age. Respondents living in a joint family were 2.67 times (1.49–4.80) more likely to have positive attitudes towards the victims of sex trafficking and anti-sex trafficking campaigns than those living in a nuclear family.

## Discussion

This is the first study to explore sex trafficking related knowledge, awareness, and attitudes among adolescent female students in Nepal, where sex trafficking is a significant problem. This study reports an above average level of awareness and positive attitude towards the victims of sex trafficking and anti-sex trafficking campaigns among adolescent female students. Some unfamiliarity and negative issues towards the victims of sex trafficking, however, still remains. In addition, the findings suggests that adolescent females’ lower socio-economic status may be associated with their lower awareness and negative attitudes towards the victims of sex trafficking and anti-sex trafficking campaigns.

Almost three-fourth of the respondents knew about sex trafficking, which indicates that students had a good basic awareness of the issue. It is noteworthy, however, that this is lower than the studies done in Nigeria (97.4%) [17], but higher than that done in Ethiopia (60%) [18]. This inter-region discrepancy may be due to the magnitude of the issue and implementation of sex trafficking prevention interventions, as well as cross-cultural variation. The sources of information about sex trafficking in this study were media (i.e. radio, television), friends, family or relatives, which is similar to the findings reported in a study conducted in Ethiopia [17]. This finding shows that mass media and social networks are playing a major role in providing relevant information. In addition, information disseminated through rallies, seminars, street theater performances, prevention camps, and community support groups are prevalent in the region [13].

Many students perceived poverty, lack of awareness, hope for a better life, and illiteracy as factors increasing the vulnerability of girls and women to trafficking. Furthermore, luring victims with false promise of good jobs and false marriage were identified at primary modes of sex trafficking. These findings are consistent with those reported in studies in South Asia, Nigeria, and South Africa [1, 5, 10, 11, 17–19], which support the claim that poverty and unemployment are the primary contributors to sex trafficking. Victims of sex trafficking are usually trafficked from relatively poorer regions to more affluent areas. Most victims were lured with promises of better jobs in areas such as India, Dubai, or Saudi Arabia [5, 10, 20, 21]. These results suggest that improving the socio-economic status of young women could be an important first step in efforts to reduce the level of sex trafficking among women in the region. Interventions designed to prevent sex trafficking need to offer programs that help women judged to be at risk for trafficking with income generation and vocational training [13].

In this study, relatives and friends were mentioned by the respondents as major mediators of sex trafficking. Previous studies conducted in Nepal have shown that some of the tactics used by these mediators include false marriages and proposals, force, and approaching indebted families to sell their daughters to pay their debts, and sometimes under the guise of a dowry for a marriage [5, 20]. The respondents also mentioned brokers as a mediator of sex trafficking, which is also consistent with other studies [17, 19, 22]. This finding suggests that brokers are increasingly operating within organized trafficking networks and using sophisticated methods. Furthermore, the majority of the students reported that young females younger than 25 years old were more vulnerable than females older than age 25. This finding is consistent with that from Nepal and other countries, which reported that women below the age of 20 years are the most-at-risk group of being trafficked [2, 18]. Prevention activities could include awareness raising and social mobilization, such as increased community surveillance, as well as improved opportunities for livelihood and the interception of suspected trafficking victims at border checkpoints. These programs can be executed through rallies, seminars, street theater performances, prevention camps, community support groups, and peer education.

Respondents in this study exhibited moderately positive attitude towards the victims of sex trafficking and anti-sex trafficking campaigns; however, there were still a significant number of respondents who had negative attitudes. These findings have important implications for anti-trafficking programs in the Nepalese context in terms of prevention programs, remediation, and advocacy. It is crucial that prevention efforts provide individuals with valuable education about trafficking and its risk factors and consequences in an attempt to reduce the likelihood that they will be trafficked, enter voluntary sex work, or migrate to pursue other work. Women and girls need to be able to feel confident that they can achieve economic prosperity within the country rather than believing that going abroad for employment is their–only option. Furthermore, various rehabilitative efforts need to be in place to help the victims of sex trafficking who are often times looked down upon and do not receive optimal care and support–such as medical checkups, counseling, job placement, etc.–as a result of stigmatization. These program should provide better life options including long-term residential and medical care, community and family advocacy, and skill development leading to gainful employment. This can achieved through non-formal education, skill and vocational training, and placement and support.

The findings from logistic regression showed that age, family type, family’s occupation type, parents educational status and having radio or TV at home were factors found associated with the respondents’ awareness and attitudes related to sex trafficking. These factors are related to availability and accessibility of information related to sex trafficking to girls and young women and how awareness on this issue changes one’s attitude [18]. This finding calls on the government, NGOs, INGOs, and other stakeholders to further prioritize actions to ensure equitable access of information regarding sex trafficking for women and girls to their raise awareness and improve attitude towards the issue.

Overall, the findings from this study highlight the importance of addressing various social determinants that are key in informing the vulnerable population about sex trafficking in Nepal. There is a compelling need for intervention that aims at raising awareness about sex trafficking among young girls and women in the rural parts of Nepal, who are at increased risk of being trafficked. The interaction of poverty and gender-based mistreatment of women and girls in families heightens the risk of sex trafficking. Therefore, prevention efforts should work to empower girls and women, improve economic opportunities and security for impoverished women and girls through including skill and development training, and formation and support of women’s cooperatives. Furthermore, interventions need to focus on educating communities regarding the tactics and identities of traffickers via awareness campaigns (including a community radio program), as well as promoting structural interventions to reduce sex trafficking.

There are several limitations to the study. First, we restricted this study to only one district (i.e., Sindhupalchowk district) and did not include out-of-school adolescent females. This limits the generalizability of the study findings to other regions and to all adolescents of a similar age group. Second, because of the self-administered questionnaire, social desirability bias may have occurred to some degree. The anonymity of the questionnaires, however, encouraged students to be honest in providing their responses. Third, we used a two-point scale to assess attitudes that may not have allowed an optimal range of responses. A five-point scale may be a better choice in our future research in this area. Despite the noted limitations, we believe this study to be an informative source of initial information regarding the problem of sex trafficking in this high risk region of Nepal.

## Conclusions

In conclusion, the majority of adolescent female students were aware about sex trafficking. The respondents had a relatively high level of knowledge and demonstrated moderately positive attitude towards the victims of sex trafficking and anti-sex trafficking campaigns. The study also revealed some key socio-demographic factors that were associated with awareness and attitudes about sex trafficking among adolescent female students. The findings presented have important implications for anti-trafficking programs, in particular those designed to educate the adolescent females who are at most-risk of sex trafficking. Educational programs need to include specific interventions to improve knowledge and attitudes towards sex trafficking among adolescent females in Nepal. Furthermore, future research could build on these findings by targeting those most-at-risk (school-attending and out-of-school women and girls) in order to substantially contribute to sex trafficking prevention and policy.
